# The Epidemiology of Antibiotic-Related Adverse Events in the Treatment of Diabetic Foot Infections: A Narrative Review of the Literature

**DOI:** 10.3390/antibiotics12040774

**Published:** 2023-04-18

**Authors:** Laura Soldevila-Boixader, Oscar Murillo, Felix W. A. Waibel, Tanja Huber, Madlaina Schöni, Rahim Lalji, Ilker Uçkay

**Affiliations:** 1 Infectious Diseases Service, IDIBELL-Hospital Universitari Bellvitge, Universitat de Barcelona, Feixa Llarga s/n, Hospitalet de Llobregat, 08907 Barcelona, Spain; 2 Research Network for Infectious Diseases (CIBERINFEC), ISCIII, 28029 Madrid, Spain; 3 Infectiology, Unit for Clinical and Applied Research, Balgrist University Hospital, 8008 Zurich, Switzerland; 4 Department of Orthopedics, Balgrist University Hospital, University of Zurich, 8008 Zurich, Switzerland; 5 Hospital Pharmacy, Balgrist University Hospital, 8008 Zurich, Switzerland; 6 EBPI-UWZH Musculoskeletal Epidemiology Research Group, Balgrist University Hospital, University of Zurich, 8008 Zurich, Switzerland; 7 Epidemiology, Biostatistics and Prevention Institute (EBPI), University of Zurich, 8008 Zurich, Switzerland; 8 University Spine Centre Zurich (UWZH), Balgrist University Hospital, University of Zurich, 8008 Zurich, Switzerland

**Keywords:** diabetic foot infection, antibiotic therapy, antibiotic-related adverse events, management

## Abstract

The use of antibiotics for the treatment of diabetic foot infections (DFIs) over an extended period of time has been shown to be associated with adverse events (AEs), whereas interactions with concomitant patient medications must also be considered. The objective of this narrative review was to summarize the most frequent and most severe AEs reported in prospective trials and observational studies at the global level in DFI. Gastrointestinal intolerances were the most frequent AEs, from 5% to 22% among all therapies; this was more common when prolonged antibiotic administration was combined with oral beta-lactam or clindamycin or a higher dose of tetracyclines. The proportion of symptomatic colitis due to *Clostridium difficile* was variable depending on the antibiotic used (0.5% to 8%). Noteworthy serious AEs included hepatotoxicity due to beta-lactams (5% to 17%) or quinolones (3%); cytopenia’s related to linezolid (5%) and beta-lactams (6%); nausea under rifampicin, and renal failure under cotrimoxazole. Skin rash was found to rarely occur and was commonly associated with the use of penicillins or cotrimoxazole. AEs from prolonged antibiotic use in patients with DFI are costly in terms of longer hospitalization or additional monitoring care and can trigger additional investigations. The best way to prevent AEs is to keep the duration of antibiotic treatment short and with the lowest dose clinically necessary.

## 1. Introduction

Diabetic foot infections (DFIs) are the major cause of non-traumatic amputations globally and are only expected to increase in absolute numbers [1]. The current management of DFIs is multidisciplinary and encompasses elements of surgery, professional wound care, off-loading, revascularization, and antibiotic administration. For many clinicians and patients and their families, the use of antibiotics for DFI represents a ‘last resort’ option before imminent amputation or surgical resection of the infection. As a result, DFIs are likely a major reason for antibiotic misuse all over the world. Even if antibiotic therapy is successful for a given DFI episode, a subsequent infection, often with a new pathogen, may appear if the underlying local (or general) cause of the former infection has not been corrected [2]. However, the clinical reliance on systemic antibiotic therapy on long-term patient outcomes may not be supported by current epidemiological evidence. 

The international IDSA and IWGDF practice guidelines provide helpful recommendations for clinicians to improve the diagnosis of infectious disease, to classify the severity of infections, to promote antibiotic stewardship in the field of DFI, and to guide antibiotic therapy [3,4,5]. However, for complete antibiotic stewardship [6], it is important to understand the epidemiology of the adverse events (AEs) of the most frequent antimicrobial agents in DFIs and the associated costs, the general economic diabetic foot costs, and the specific AE profile of an antimicrobial agent, which can contribute significantly to increased costs in higher monitoring care and additional days of hospitalization [7,8]. This is of particular importance when clinicians are not entirely convinced of the benefits of long-lasting antibiotic treatment and when the longer antibiotics are associated with the increase in multidrug-resistant microorganisms [9]. Moreover, we are aware of the vulnerable nature of comorbid DFI patients to AEs and interactions with concomitant medications [10]. 

In order to fill these clinical knowledge gaps, we performed a narrative review with an epidemiological focus, with the main aim to summarize the most frequent and most severe AEs reported in prospective trials and observational studies at the global level in DFI. 

### 1.1. General Principles for the Use of Antibiotics in Diabetic Foot Infection

Systemic antibiotics administered orally or intravenously have been shown to be effective in the treatment of DFIs; however, none have been shown to be superior to others [11]. The choice and duration of antibiotic therapy depends on several clinical considerations, such as the severity of the infection, soft tissue versus bone infection, and surgical versus conservative treatment of the infection [5]. According to practice guidelines, for mild soft-tissue DFIs an antibiotic duration of 7–10 days is appropriate [12]. However, this can be prolonged for up to 3–4 weeks in cases of severe infection. For example, diabetic foot osteomyelitis (DFO) typically requires a prolonged duration of treatment. In cases with residual infected bone after (partial) amputation, the post-surgical duration is set at 2–4 weeks. In strictly conservative treatment without surgery, the current recommended duration is 4–6 weeks [5]. Interestingly, recommendations that describe the duration of antibiotic prescription before surgical debridement are censored, and the recommendations of duration that apply for the postoperative period are under debate. Likewise, the concomitant use of topical or of intraosseous antibiotic administrations does not alter the recommendations for the simultaneous systemic antibiotic duration in the DFO setting.

### 1.2. Choice of Antibiotic Agents

Clinicians should individualize the choice of antibiotic agent based on the pathogen, its susceptibilities, patient comorbidities, potential drug–drug interactions, and contraindications. Although it is beyond the scope of this focused manuscript to report on all possible drug–drug interactions, Supplementary Table S1 provides an overview of the most relevant interactions in the therapy of patients with DFI [13].

Beta-lactams are the most frequently used antibiotic class in DFIs for parenteral and/or empirical therapy [14]. The polymicrobial etiology of DFI, with Gram-positive cocci as the main protagonists and others including enterococci [15] and anaerobes, can be covered with the use of amoxicillin/clavulanate (A/C) and piperacillin/tazobactam (P/T), the latter also treated against non-fermenting rods such as *Pseudomonas aeruginosa*. However, these antibiotics do not cover bacteria stemming from skin commensals in DFO or methicillin-resistant *Staphylococcus aureus* (MRSA) [16]. In acute and severe infections, the use of P/T and carbapenems may cover almost all bacteria. Regarding the less virulent skin commensal Gram-positive bacteria in chronic DFO, including for MRSA and vancomycin-resistant enterococci, clinicians typically refer to glycopeptides, lipopeptides, and lipoglycopeptides (e.g., vancomycin, teicoplanin, daptomycin, dalbavancin, oritavancin) or the oxazolidinone family of antibiotics (linezolid and tedizolid) for treatment. 

## 2. Results

### 2.1. Literature Search

#### 2.1.1. General Findings

We first performed a literature search in the English language on the PubMed database for relevant prospective trials and observational studies related to DFIs. During the first search, the terms “diabetic foot” and “antibiotics” were used. This search retrieved 144 articles, with 22 studies selected for full text review. A second search was conducted using terms related to “soft tissue infection” or “skin-structure infection and antibiotics”, which resulted 1151 potentially relevant articles. Of these, 264 articles underwent full text analysis and 29 were included in this review. Two additional articles were found through non-electronic methods and included in this review. In total, we included 53 studies. Details of the articles are shown in Table 1 with information on AEs expressed in percentages during and/or after systemic antibiotic therapy. The percentage was calculated as a proportion of the number of adverse reactions associated with the study antibiotics in relation to the total number of patients in the study group.

From these included articles, we found the incidence of AEs varied between 2% and 30% for patients with DFI. The most common AEs were gastrointestinal in nature, covering 5% to 22% of all reported AEs. In the following text, we summarize the most common AEs in patients with DFI stratified against a variety of antibiotic classes through the accurate revision of the selected articles.

#### 2.1.2. Penicillins

AEs ranged from the lowest 2.8% (95% confidence interval, 95% CI 2–4%) [17] to the highest 22% (95% CI 17–29%) [18] from the penicillin or penicillin derivatives group. Skin reactions for various (amino)penicillins were described at low frequencies (1–3%), whereas anaphylaxis or systemic reactions were shown to be rare [12,19] among DFI patients. Gastrointestinal AEs were shown to be the most common and range from 4.5% with amoxicillin/clavulanate (A/C) [20] to between 4.1% and 6.5% with ten days of ticarcillin/clavulanate (T/C) or P/T treatment [21]. A higher proportion of AEs (14%) was found in studies with ampicillin/sulbactam (A/S) with prolonged administration of 10 to 22 days and with P/T for 17 days [22,23]. Additionally, we found 22% for ticarcillin/clavulanate (T/C) followed by A/C for 14 days [18]. In three studies, symptomatic *Clostridium difficile colitis* (CDI) was reported more frequently during beta-lactam therapy compared with other groups of antibiotic classes (0.5% for non-beta-lactam antibiotics [24] and 2% to 8–9% for beta-lactams such as A/S or I/C, respectively [25,26]). Other AEs reported under penicillin treatment are cytopenia, leucopenia, and thrombocytopenia, which range from 0.6% to 6.7% [27,28]. A symptomatic hepatotoxicity was described between 3% and 16.7%, with higher proportions reported during P/T treatment [28,29]. The high sodium content within intravenous solutions of some beta-lactams such as P/T can initiate decompensated cardiac failure [12]. In addition, penicillins used in combination with fosfomycin may increase the risk for gastrointestinal AEs [30].

#### 2.1.3. Cephalosporins

With regards to cephalosporins, only a small number of AEs are reported in the DFI literature. Diarrhea appears to occur less frequently with cephalosporins compared with other beta-lactams (6% vs. 9%; respectively). Skin rash (1%) and hepatoxicity (0.5%) [31] were shown to be rare in contrast to other AEs, particularly with parenteral cephalosporins (including the newer generations, ceftobiprole and ceftaroline), which may produce increased nausea, diarrhea, headache, and generalized pruritus (5.7%, 3.4%, 5.1%, and 3.7%, respectively); CDI was diagnosed in 0.6% [32,33,34]. Ceftobiprole showed higher percentages of nausea (11%) [35].

#### 2.1.4. Carbapenems

Gastrointestinal perturbances were found as the most common AE occurring in between 3% and 12% of cases for imipenem/cilastatine (I/C) or meropenem treatments. Symptomatic CDI occurred at a rate of 8 to 9% during I/C treatment [25,36]. Hepatotoxicity was described to be up to 10% with ertapenem [37]. Seizures, a severe AE, were described with carbapenems (meropenem and I/C) in 0.2% of patients [38] and were specifically slightly higher with I/C, from 2% to 2.2% [25,26]. 

#### 2.1.5. Fluoroquinolones

Although gastrointestinal AEs are also the most frequently reported, quinolones show fewer AEs related to diarrhea when compared with beta-lactam therapy (4 to 5%) [39,40]. Rarer AEs include hepatotoxicity, interstitial nephritis, or deliriant central nervous system effects [41,42]. In a clinical trial, ofloxacin showed neurological symptoms in 16% of cases (i.e., headache and insomnia) after a mean duration of 21 days after starting treatment [43]. Pefloxacin yielded a global risk for gastrointestinal AEs of 8.8% [44]. Sparfloxacin presented symptomatic photosensitivity in 11% of cases, whereas this was shown to occur in 2% of cases with clinafloxacin and 0.7% with ciprofloxacin [45,46]. An asymptomatic prolongation of the QTc time was observed for sparfloxacin in 0.5% to 2.4% of the therapeutic episodes [24,47]. An asymptomatic hepatitis due to fluoroquinolones was shown to occur in 0.2% to 3.2% of cases [24,48]. In 2018, the Food and Drug Administration (FDA) warned about possible fluoroquinolone-associated ruptures of pre-existing aortic aneurysms. Since then, other reports have echoed a similar claim [49]. The current recommendation is to avoid quinolones in cases of severe tendinopathies or aortic aneurisms. However, we did not identify any reports on aneurism ruptures related to the use of quinolones specifically in the DFI population. Likewise, despite an abundance of foot and ankle pathologies in adult diabetic populations, no reports of increased Achilles tendon disease were found in the DFI population. Newer quinolones, such as delafloxacin and levonadifloxacin, with broad-spectrum antimicrobial activity (including for MRSA strains) showed a low risk for AEs. For example, hepatic alterations occurred in 3.2% of patients and hyper/hypoglycemia was shown to occur in between 0.1% and 0.3% of cases for delafloxacin, and gastrointestinal symptoms occurred in 5.2% of cases for levonadifloxacin [48,50].

#### 2.1.6. Rifampin/Rifampicin

In a landmark randomized clinical trial regarding the duration of antibiotic therapy for the conservative management of DFO, 27 (68%) of the DFI patients received rifampicin and 19 (48%) received rifampicin with levofloxacin. In this study, antibiotic-related adverse events were recorded in 16 patients (40%); 6 patients (30%) at 6 weeks of follow-up and 10 patients (50%) at the 12-week follow-up timepoint. The most common AEs found were gastrointestinal (nausea and vomiting), all attributable to rifampicin at the 12-week follow-up timepoint (10%) [51]. Rifampin also has potential for significant drug interactions in addition to these AEs (see Supplemental Table S1). 

#### 2.1.7. Glycopeptides, Lipopeptides, and Lipoglycopeptides

Glycopeptides such as vancomycin are commonly associated with hypersensitivity reactions such as infusion reaction and rash, known as red-man syndrome, which is an expected histamine release with immediate skin rash (3–40%) [52,53]. Infusion reactions have also less been described with telavancin, dalbavancin, and oritavancin. Other true AEs may include vancomycin nephrotoxicity [54,55] or elevated serum creatin-phosphokinase or rhabdomyolysis with daptomycin (2.8–3%) [56,57]. Data on newer lipoglycopeptide drugs indicate that dalbavancin showed reduced AEs in soft-tissue infections [58], with less nausea, diarrhea, and pruritus [59,60]. Many of these relatively new drugs are not commonly used in DFI; however, experts expect that their use will increase in the coming decades. Of note, teicoplanin is not available in every country.

#### 2.1.8. Oxazolidinones: Linezolid and Tedizolid

These agents, both with nearly universal anti-Gram-positive activity, may cause potentially substantial toxicities (transient myelosuppression or lactic acidosis and optic or peripheral neuropathy) due to impaired mitochondrial protein synthesis and consecutive mitochondrial dysfunction. A common feature is the expected drop in serum platelet and hemoglobulin counts, which usually occurs two weeks after the start of treatment [52,54]. Linezolid, compared with other oxazolidinones, generally results in anemia and thrombocytopenia that is entirely related to the duration of treatment. Patients who developed anemia after linezolid typically did so when treatment lasted more than 14 days [61]. Additionally, clinical trials comparing tedizolid with linezolid revealed reduced thrombocytopenia (0% vs. 4.9%) and neutropenia–pancytopenia (0% vs. 2.4%) from tedizolid after an average duration of 10 days [62]. 

#### 2.1.9. Aminoglycosides

Our search revealed that no clinical trials or multicenter studies with the use of an aminoglycoside as a systemic antibiotic for DFIs were published. However, gentamicin was the most frequently used topical antibiotic agent for infected diabetic foot ulcers and also takes place as intraosseous deposits for chronic osteomyelitis in the diabetic foot and other orthopedic infections. When used as gentamicin sponges for infected diabetic foot ulcers, gentamycin showed no systemic AE and was very well tolerated [63,64]. However, the use of any topical antibiotic treatment is not yet recommended in international guidelines, at least not as a combination with systemic antimicrobial agents [5].

#### 2.1.10. Tetracyclines 

Gastrointestinal symptoms are the most common AE in this antibiotic group. According to reports captured through our search, nausea and vomiting may occur up to 25% (95% CI, 19–32%), 35% (95% CI, 31–39%), or 40% (95% CI. 36–45%) of the time [65,66,67]. Furthermore, the newer tetracyclines (omadacycline) showed greater gastrointestinal tolerance at standard doses. However, a higher loading dose was still associated with nausea and vomiting [68]. Few studies showed lower percentages of gastrointestinal events (less than 2%) when the duration of treatment and the prescription of other antibiotics was at the discretion of the physician [69]. 

#### 2.1.11. Cotrimoxazole

As widely experienced by many clinicians, some articles confirmed the potential for a worsening of transient kidney function in the laboratory with the combination of cotrimoxazole and cephalexin [70]. Other risks are seldom, such as skin rash or hepatitis.

#### 2.1.12. Clindamycin

The most common AEs are gastrointestinal in nature, with few reported cases of pseudomembranous colitis due to *Clostridium difficile*. We found that the relation of CDI with clindamycin in monotherapy was around 1.7% for an average duration of 14 days [71]. 

**Table 1:** Adverse events related to the use of antibiotics in diabetic foot infections (soft tissue, osteomyelitis, and wound infection).

Study Details	Study Design	Antibiotic, Dosage	Duration of Antibiotics	Target Group	Adverse Events (AE) Related to Antibiotic (Binomial Exact Confidence Interval, CI 95%)
[71] Lipsky, 1990 USA	RCT	**A:** Clindamycin 300 mg/6 h vs. **B:** Cephalexin 500 mg/6 h	14 days	N = 56 DFI, A: 27 vs. B: 29	-Overall, 5% (1–15%) of GI AE. -CDI was 1.7% (0–10%) in A. -Nausea and diarrhea were 3.6% (0–12%) in B.
[44] Segev, 1990 Israel	RCT	**A:** Pefloxacin 400 mg/8 h and then every 12 h vs. **B:** Ceftazidime 2 g/12 h	13 days vs. 10 days	N = 67 STI, A: 34 vs. B: 33 (33 diabetic infections)	-Nausea and vomiting were 8.8% (19–24%) in A.
[21] Tan, 1993 USA, Canada	RCT	**A:** P/T 3 g/6 h vs. **B:** T/C 3 g/6 h	10 days	N = 251, A: 153 vs. B: 98 (63 diabetic infections)	-GI were present of 11% in each treatment group (A: 7–17% vs. B: 6–19%). Diarrhea was the most common, A: 6.5% (3–12%) vs. B: 4.1% (1–10%).
[26] Grayson, 1994 UK	RCT	**A:** I/C 500 mg/6 h vs. **B:** A/S 3 g/6 h	12–13 days	N = 96 DFI, 48 in each arm	-Overall, 16.7% (10–26%) without differences between the groups. -Diarrhea was 8.3% (2–20%) with 2% (0–11%) CDI in B. -Diarrhea was 10.4% (3–23%) with 8% (2–20%) CDI in A. -Seizures were 2% (0–11%) in A.
[31] Schwartz, 1996 USA	RCT	**A:** Cefepime 1 g/12 h vs. **B:** Ceftazidime 1 g/8 h	3–18 days vs. 4–16 days	N = 298 STI, A: 198 vs. B: 100 (91 diabetic infections)	-Overall, 15% (11–20%), without differences between the groups. -GI were 6% (3–10%) in A vs. 9% (4–16%) in B, headache was 5% in A vs. 1% in B, rash 1% in A vs. 1% in B. -Liver alteration (0.5%) with cefepime that improved with stopping treatment.
[20] Chantelau, 1996 Germany	RCT	**A:** Placebo vs. **B:** A/C 500 mg/125 mg/8 h	Not specified.	N = 44 DFU, A: 22 vs. B: 22	-Self-limiting diarrhea was 4.5% (0–23%) in B.
[22] Akova, 1996 Turkey	IT	A/S 1.5 g/6 h	10–22 days for soft tissue and 27–56 for DFO	N = 74 DFI	-Diarrhea was 14% (7–23%).
[25] McKinnon, 1997 USA	RCT	**A:** A/S 3 g/6 h vs. **B:** I/C 500 mg/6 h	13 vs. 15	N = 90 DFI, 45 each group	-AEs were 16% (6–29%) in A vs. 20% (10–35%) in B. -Diarrhea related to CDI was 2.2% (0–12%) in A vs. 8.9% (3–21%) in B. -Seizures were 2.2% (0–12%) in B.
[43] Lipsky, 1997 USA	RCT	**A:** Ofloxacin 400 mg/12 h vs. **B:** A/S 1–2 g/6 h and after A/C 500 mg/8 h	21 days	N = 108 DFI, 55 vs. 53	-Overall, 31% (19–45%) in A, including 16% (8–29%) of neurological symptoms (headache, insomnia) and 11% (4–22%) of GI. -17% in B, including 7.5% (2–18%) miscellaneous and 5.7% (1–16%) GI.
[46] Lipsky, 1999 USA	RCT	**A:** Sparfloxacin 200 mg/12 h or **B:** Ciprofloxacin 750 mg/12 h	10 days	N = 603 STI, 298 vs. 305 (111 diabetic infections)	-Overall, 6% (4–9%) in A and 23% (18–28%) in B. -GI occurred in 7.1% (4–11%) in A vs. 19% (15–24%) in B. -Photosensitivity reactions, 11% (8–15%) in A vs. 0.7% (0–2%) in B. -QTc interval from baseline to the maximum value was greater in the sparfloxacin group. *Not specified the values*.
[45] Siami, 2002 USA	RCT	**A:** Clinafloxacin 200 mg/12 h vs. **B:** P/T 3 g/6 h	13 days	N = 409 STI, 213 vs. 196 (76 diabetic infections)	-Drug-related AEs were 7.5% (4–12%) in A vs. 5% (2–9%) in B. -Photosensitivity reaction was 1.9% (1–5%) in A. -Diarrhea was 0.5% (0–3%) in A and 2.6% (1–6%) in B.
[29] Graham, 2002 USA and International collaboration	RCT	**A:** Ertapenem 1 g daily vs. **B:** P/T 3.375 g/6 h	9–10 days	N = 540 STI, 274 vs. 266 (98 diabetic infections)	-Overall, 24% (19–30%) in A vs. 23% (18–28%) in B. -Diarrhea was 5.5% (3–9%) in A and 9% (6–13%) in B, nausea 3.7% (2–7%) in A and 2.7% (1–5%) in B, and rash 1.8% (0.5–4%) in A and 1.2% (0–3%) in B. -The alteration of liver function (high ALT, AST, or alkaline phosphatase) was 8.4% (5–12%) in A and 8.6% (6–13%) in B.
[18] Graham, 2002 USA	RCT	**A:** Levofloxacin 750 mg daily vs. **B:** T/C 3.1 g/4–6 h, followed by A/C 875 mg/12 h	7–14 days	N = 399 STI, 200 vs. 199 (67 diabetic infections)	-GI was the most common, 18.5% (13–25%) in A vs. 22.5% (17–29%) in B. -Other: 1% dysuria in A and 1% serum sickness and genital pruritus in B.
[56] Arbeit, 2004 USA	RCT	**A:** Daptomycin 4 mg/kg daily vs. **B:** penicillin derivative 4–12 g per day or vancomycin 1 g/12 h	7 days	N = 902 STI, 534 vs. 558 (133 diabetic infections)	-Overall, 18% (15–22%) in A vs. 21% (18–0.25%) in B. -Diarrhea, 5.2% (4–7%) in A vs. 4.3% (3–6%) in B. -Elevations in CPK levels were reported in 2.8% (2–5%) in A and only 1.8% (1–3%) in the comparator group.
[61] Lipsky, 2004 USA and International collaboration	RCT	**A:** Linezolid 600 mg/12 h vs. **B:** A/S (1.5–3 g/6 h iv) or A/C (500 mg/8–12 h).	16 days vs. 15 days	N = 361 DFI, 241 vs. 120	-A was associated with a 16% increase in the rate of adverse events compared with B. -Diarrhea was 7.5% (4–12%) in A vs. 3.3% (1–8%) in B; nausea 5.8% (3–10%) in A vs. 0% in B; anemia and thrombocytopenia 4.6% (2–8%) and 3.7% (2–7%) in A vs. 0% in B.
[27] Harkless, 2005 USA	RCT	**A:** P/T 4 g/8 h vs. **B:** A/S/6 h	8 days	N = 314 DFI, 155 vs. 159	-Overall, 18.7% (13–26%) in A vs. 13.2% (8–19%) in B. -Diarrhea 7.1% (4–12%) in A vs. 2.5% (1–6%) in B; nausea 3.2% (1–7%) in A vs. 0.6% (0–3%) in B. -Leukopenia and thrombocytopenia 0.6% in A.
[57] Lipsky, 2005 USA, Europe, South Africa, Australia, and Israel	RCT	**A:** Daptomycin 4 mg/kg/24 h vs. **B:** Vancomycin 1 g/12 h or semisynthetic penicillin	7–14 days	N = 133 DFI, 61 vs. 72	-Overall, there were fewer adverse events in A compared with the other antibiotics. -GI events were the most frequent. Diarrhea 3.3% (0–11%) in A vs. 2.8% (0–10%) in B. -Elevated CPK during the second week of treatment, 3% (0–11%) in A.
[23] Lipsky, 2005 USA	RCT	**A:** Ertapenem 1 g daily vs. **B:** P/T 3.3.75 g/6 h, minimum 5 days. After, A/C (875/125 mg/12 h) could be given.	11 days for IV treatment; 17 days total treatment	N = 576 DFI, 289 vs. 287	-Overall, 15% (11–20%) in A vs. 20% (15–25%) in B. -Diarrhea, 8% (5–12%) in A vs. 14% (10–19%) in B; nausea 6% (3–9%) in A vs. 7% (4–10%) in B; headache 3.5% (2–6%) in A vs. 6% (3–9%) in B. -Laboratory events 4% (2–7%) vs. 10% (6–13%). *No other details*.
[30] Stengel, 2005 Germany	RCT	Fosfomycin 8 g to 24 g daily plus a combination of beta-lactam	14 days	N = 52 DFI	-Overall, 7.7% (2–19%) of nausea and rash.
[52] Itani, 2005 USA	RCT	**A:** Linezolid 600 mg/12 h vs. **B:** vancomycin 1 g/12 h.	12 vs. 11 days	N = 1180 diabetic patients, 592 vs. 588	-Overall, 22% (19–26%) in A vs. 21% (17–24%) in B. -Diarrhea was 5.2% (4–7%) in A vs. 1.5% (1–3%) in B and nausea 4.1% (3–6%) in A vs. 1.4% (1–3%) in B. -Thrombocytopenia was 3.5% (2–5%) only in A. -Rash was 0.5% (0–1%) in A vs. 2.7% (2–4%) in B.
[38] Fabian, 2005 USA	RCT	**A:** Meropenem 500 mg/8 h vs. **B:** I/C 500 mg/8 h. Switch to an oral option on the third day.	9 days	N = 1037 STI, 511 vs. 526 (95 diabetic patients)	-Overall, 4.3% (3–6%) in A vs. 7.2% (5–10%) in B. -GI (diarrhea, nausea, constipation) 3.1% (2–5%) in A vs. 5% (3–7%) in B; headache 0.6% in both groups; pruritus 0.4% in A vs. 1.5% in B. -0.2% of seizures were noticed in both groups.
[39] Giordano, 2005 USA	RCT	**A:** Moxifloxacin 400 mg daily vs. **B:** P/T 3 g/6 h, followed by A/C 800 mg/12 h	6–14 days	N = 601 STI, 298 vs. 303 (50 diabetic patients)	-Overall, 31% (26–37%) in A vs. 30% (25–36%) in B. -Diarrhea was 5% (3–9%) in A vs. 8% (5–12%) in B.
[66] Ellis-Grosse, 2005 USA	RCT	**A:** Tigecycline 100 mg followed by 50 mg/12 h vs. **B:** vancomycin 1 g/12 h plus aztreonam 2 g/12 h	8 days	N = 1116 STI, 566 vs. 550 (168 diabetic patients)	-GI manifestations were more frequent in A, nausea being 34.5% (31–39%) in A vs. 8.2% (6–11%) in B and vomiting 19.6% (16–23%) vs. 3.6% (2–6%), respectively. -Rash was 1.9% (1–3%) in A vs. 5.8% (4–8%) in B and the increase in AST was 1.8% (1–3%) in A vs. 5.1% (3–7%) in B.
[36] Embil, 2006 Canada	RCT	**A:** Meropenem 500 mg/8 h vs. **B:** I/C 500 mg/8 h	7–14 days	N = 398 DFI, 204 vs. 194	-GI problem 11.8% (8–17%) in A vs. 7.2% (4–12%) in B and headache 9.8% (6–15%) in A vs. 6.2% (3–11%) in B.
[42] Lipsky, 2007 USA	RCT	**A:** Moxifloxacin 400 mg/day vs. **B:** P/T 3 g/6 h, followed by oral A/C 800 mg/12 h	6–7 days	N = 617 STI, 306 vs. 311 (110 diabetic infections)	-Overall, 31.7% (27–37%) in A and 13% (9–17%) in B. -Diarrhea 12.7% (9–17%) in A vs. 9.4% (6–13%) in B; headache 4.8% (3–8%) and pruritus 4.8% (3–8%) in A.
[35] Noel, 2008 USA	RCT	**A:** Ceftobiprole 500 mg/12 h vs. **B:** vancomycin 1 g/12 h	9 days	N = 822 STI, 543 vs. 279 (302 diabetic infections)	-Nausea, 11% (8–14%) in A and 6% (4–10%) in B.
[65] Teras, 2008 Multicenter, Europe	RCT	**A:** Initial dose of tigecycline 100 mg followed by 50 mg/12 h vs. **B:** vancomycin 1 g/12 h plus aztreonam 2 g/12 h	14 days	N = 376, 189 vs. 187 (53 diabetic patients)	-Nausea and vomiting, 25% (19–32%) in A vs. 12% (8–17%) in B. -The increase in AST and ALT liver enzyme was the most reported in B, 5%-7% (4–11%) vs. 2% (1–5%) in A.
[40] Vick-Fragoso, 2009 International	RCT	**A:** Moxifloxacin 400 mg daily vs. **B:** A/C 1 g/500 mg/8 h	14 days	N = 804 STI, 406 vs. 398 (134 diabetic infections)	-Overall, AEs were 18% (14–22%) in A vs. 16% (13–20%) in B. -The most common AEs leading to withdrawal from the study in A were 4% for GI and cutaneous manifestation. -The drug-related adverse events leading to withdrawal were GI (6%) and cutaneous manifestation (1.5%) in B.
[28] Saltoglu, 2010 Turkey	RCT	**A:** P/T 4.5 g/8 h or **B:** I/C 0.5 g/6 h.	21 vs. 24 days	N = 62 DFI, 30 vs. 32	-AEs were 29% (15–49%) in A compared with 9% (2–25%) in B. -Hepatoxicity was 16.7% (6–35%) in A vs. 3.1% (0–16%) in B, nephrotoxicity 20% (8–39%) in A vs. 3.1% (0–16%) in B, and hematological side effects 6.7% (1–22%) in A (*not specified the type*).
[54] Itani, 2010 International	RCT	**A:** Linezolid 600 mg/12 h or **B:** vancomycin 15 mg/kg/12 h	9 vs. 8 days	N = 1052, 537 vs. 515 (106 diabetic infections)	-AEs were 23% (20–27%) in A vs. 22% (18–26%) in B. -All treatment-related hematologic adverse events were low in both treatment arms but occurred more often in the A group (*not n; % specified*). -In contrast, treatment-related nephrotoxic adverse events occurred more frequently in the B group (*not n; % specified*).
[32] Corey, 2010 International	RCT	**A:** Ceftaroline fosamil 600 mg/12 h ± placebo/12 h vs. **B:** Vancomycin 1 g/12 h plus aztreonam 1 g/12 h	7 days	N = 702 STI, 353 vs. 349 (130 diabetic infections)	-Nausea 5.7% (3.5–8.6%) in A and 4.6% (3–7%) in B, headache 5.1% (3–8%) in A and 3.7% (2–6%) in B, and generalized pruritus 3.7% (2–6%) in A vs. 4.6% (3–7%) in B. -Diarrhea 3.4% (2–6%) in A with 2 CDI (0.6%) in A.
[24] Gyssens, 2011 International	RCT	**A:** Moxifloxacin 400 mg daily vs. **B:** P/T 4 g/8 h followed by oral A/C 875/12 h	7–14 days	N = 813 STI, 432 vs. 381 (269 diabetic infections)	-Overall, 8.7% (6–12%) in A vs. 7.4% (5–10%) in B. -Diarrhea, 1.9% (1–4%) in A vs. 1.1% (1–3%) in B, 0.5% CDI in general. -Serious drug-related AEs in B were prolonged QT interval (0.5%) and increased blood alkaline phosphatase (0.2%).
[53] Chuang, 2011 India, Taiwan	RCT	**A:** 100 mg initial dose of tigecycline and 50 mg/12 h vs. **B:** vancomycin 1 g/12 h–aztreonam 2 g/12 h	7 days Indian patients; 11 days Taiwanese patients	N = 128 STI, (45 diabetic infections)	-In the Indian population, the most common AE was GI, nausea and/or vomiting 40.9% vs. 11.9%, *p* = 0.003. The abnormality of the coagulation system included the prolongation of the prothrombin time (27.3% vs. 7.1%, *p* = 0.02) and the prolonged activation of the partial thromboplastin time (38.6% vs. 14.3%, *p* = 0.02). -In the Taiwanese population, nausea and/or vomiting occurred in 52% vs. 9.1%, *p* = 0.005. More patients treated with V/A had rash or pruritus (41% vs. 26%) without a statistically significant difference.
[47] Schaper, 2013 The Netherlands	RCT	**A:** Moxifloxacin 400 mg daily or **B:** P/T 4 g/8 h followed by oral A/C 875 mg/12 h.	14 days	N = 206 DFI, 110 vs. 96	-Overall, 31% (22–40%) in A vs. 32% (23–43%) in B. -The most common were GI (A:2.4% vs. B:6.3%). -QT electrocardiogram prolonged (A:2.4% vs. B:0.9%). -Hypertension (A:4.1% vs. B:0.9%); insomnia (A:2.4% vs. B:1.8%).
[41] Bogner, 2013 International	Obs	Moxifloxacin 400 mg daily IV or oral.	10–11 days	N = 5444 STI (1730 diabetic infections)	Overall, the AEs were low, 2.6% (2–3%). -GI events were the most frequent (diarrhea and nausea), which affected 27 (0.5%) and 21 (0.39%) of patients, respectively. -Adverse central nervous system events such as headache and dizziness were the next most frequent, affecting 0.18% and 0.15% of patients.
[33] Santos, 2013 USA	RS	Ceftaroline (dose not specified)	6 days	N = 647 STI (295 diabetic infections)	-2% (1–3%) of AEs, *details not specified*.
[69] Montravers, 2013 Europe	Obs	Tigecycline (dosage, duration of treatment was at the discretion of the physician)	12 days	N = 254 STI (126 diabetic infections)	-Nausea and vomiting in ≤2% of patients. -The most common serious AEs were multi-organ failure, in 4% (2–7%)
[17] Garau, 2013 Europe	Obs	Several antibiotics (penicillins and quinolones)	14.6 days	N = 1995 (237 diabetic infections)	-Overall, the AEs were low, 2.8% (2–4%).
[67] Laszlo L, 2014 International	RCT	**A:** 150 mg of tigecycline once daily vs. **B:** 1 g of ertapenem 1 g vancomycin once daily	25 days vs. 39 days	N = 944 DFI, 477 vs. 467	-Nausea–vomiting was 40% (36–45%) in A vs. 25% (21–29%) in B. -The AEs leading to discontinuation of tigecycline were primarily nausea (2.7%) and vomiting (2.3%); these occurred significantly more frequently than in subjects treated with ertapenem ± vancomycin (*p* = 0.01 and *p* = 0.001, respectively).
[59] Boucher, 2014 International	RCT	**A:** Dalbavancin 1 g administered intravenously on day 1, followed by 500 mg on day 8 vs. **B:** vancomycin 1 g/12 h for at least 3 days, with the option to switch to oral linezolid 600/12 h	10–14 days	N = 1303, 652 vs. 651 (147 diabetic infections)	-Overall, 12% (10–15%) in A vs. 14% (11–17%) in B. -The most common AEs were nausea 2.5% in A and 2.9% in B, diarrhea 0.8% in A and 2.5% in B (*p* = 0.02), and pruritus 0.6% in A and 2.3% in B (*p* = 0.01).
[34] Lipsky, 2015 USA	RS	Ceftaroline 600 mg/12 h	6.1 days	N = 201 DFI	-2% (1–5%) discontinued the treatment because of an adverse event. (*Acute myocardial infarction in 1 out of 4; not recorded for the other three patients*)
[51] Tone, 2015 France	RCT	Most commonly used antibiotics fluoroquinolones +/− rifampicin	12 weeks vs. 6 weeks	N = 40 DFO, 20 vs. 20	-Nausea, vomiting, diarrhea, and liver cytolysis/cholestasis were more common in 12-week treatment than in 6-week treatment (10%, 10%, 10%, 15% vs. 5%, 5%, 0%, 5%).
[60] Corey, 2015 International	RCT	**A:** Oritavancin 1200 mg single dose vs. **B:** vancomycin 1 g/12 h.	8.4 days	N = 1005 STI, 503 and 502 (91 diabetic infections)	-AEs were low, 3.6% (2–6%) in A vs. 2.6% (1–4%) in B. -A vs. B: Pruritus 2.6 vs. 5.8%, diarrhea 2.6 vs. 3%, AST increased 2.2 vs. 2.2%, dizziness 2.2 vs. 2.2%.
[37] Zhang-Rong Xu, 2016 China	RCT	**A:** Ertapenem 1 g daily or **B:** P/T 4.5 g/8 h	10 vs. 12 days	N = 550 DFI, 275 in all arms	-5% (3–7%) of patients have at least one side event. -GI were the most common, 9.1% (6–13%) in A vs. 7.6% (5–11%) in B. -Dizziness 2.5% in A vs. 1.8% in B. -ALT increased (≥3× upper limit of normal) did not differ significantly between the two groups (10.2% vs. 4.4%).
[70] Moran, 2017 USA	RCT	**A:** Cephalexin (one 500-mg pill 4 times daily) plus placebo (4 pills twice daily) vs. **B:** Cephalexin plus T/S (4 single-strength pills, 80 mg/400 mg, twice daily).	7 days	N = 496 STI, 248 in every arm (45 diabetic infections)	-The most common GI, 38.7% (33–45%) in A and 46% (40–53%) in B. (*One case of CDI attributed to clindamycin administered after treatment failure occurred in B*). -1 severe AE occurred in B (acute-on-chronic kidney injury resolved). -Severe adverse events were not significantly different, 0.4% in A vs. 1.2% in B.
[62] Mikamo, 2018 Japan	RCT	**A:** Tedizolid 200 mg daily vs. **B:** Linezolid 600 mg/12 h	10 days for both	N = 125 STI, 84 vs. 41 (58 diabetic infections)	-GI disorders were 2.4% (0–8%) in A vs. 12% (4–26%) in B. -Higher thrombocytopenia with linezolid 0% in A vs. 4.9% in B, also neutropenia and pancytopenia 0% in A vs. 2.4% in B.
[48] Bassetti, 2019 International	RCT	**A:** Delafloxacin at 300-mg IV and 450-mg oral tablet vs. **B:** Vancomycin 15 mg/kg plus aztreonam 1–2 g/12 h	6 days (1–14)	N = 741 vs. 751 STI (167 diabetic patients)	-Hepatic events, 3.2% (2–5%) in A vs. 3.4% (2–5%) in B. -Rates of treatment-related hyperglycemia and hypoglycemia were similar between patients in A vs. B: 0.3% vs. 0.1% and 0.1% vs. 0.3%, respectively. -CDI 0.1% in A (*previous treatment with T/S and clindamycin*). -No cases in the delafloxacin group with QT prolongation or convulsions.
[58] Rappo, 2019 International	RCT	Dalbavancin 1500 mg intravenously as a single dose or two doses (1000 mg followed by 500 mg a week later).	-	N = 698 STI N = 76 diabetic patients	-Treatment-emergent adverse events occurred in 23.7% of outpatients receiving a single dose and in 21.2% of outpatients receiving the two-dose regimen. -Serious AEs were low (1.3–2.6%) but not described.
[68] Abrahamian, 2019 USA	RCT	**A:** Omadacycline 100 mg IV/12 h for 2 doses, then 100 mg IV daily for 2 days vs. **B:** linezolid 600 mg/12 h	8.7 days	N = 1380 STI (108 diabetic patients)	-GI adverse events were the most common. -Oral administration of A was associated with higher rates of GI events (nausea and vomiting), associated with the loading dose of 450 mg during the first 2 days of oral only, and the rates decreased thereafter.
[55] Trinh, 2019 USA	RCT	**A:** Ceftaroline 600 mg/12 h vs. **B:** vancomycin 1 g/12 h	4–6 days	N = 724 STI, 325 vs. 399 (274 diabetic patients)	-Nephrotoxicity was rare overall (1.5%), although there was a numerically higher rate observed in the vancomycin group (2.2% vs. 0.3%, *p* = 0.08). -No documented cases of *Clostridioides difficile*-related diarrhea or serious AEs.
[50] Bhatia, 2020 USA	RCT	**A:** Levonadifloxacin 1000 mg/12 h vs. **B:** Linezolid 600 mg/12 h	7–10 days	N = 500 STI, 250 including diabetic patients	-GI were 5.2% (3–9%) in A vs. 6% (3–10%) in B. -Hemoglobin decreased by 0.8% in A vs. 1.2% in B.
[19] Gariani, 2021 Switzerland	RCT	Several antibiotics	3 weeks vs. 6 weeks	N = 93 DFO	-The number of AEs was similar between the groups. -Overall, 12% (6–20%) AEs (4% fungal intertrigo, 1% anaphylaxis to A/C, 1% drug fever with cotrimoxazole, 3% skin rash to amoxicillin or levofloxacin, 1% severe diarrhea due to levofloxacin, and 1% persistent nausea with clindamycin).
[12] Pham, 2022 Switzerland	RCT	Co-amoxiclav (45), levofloxacin (13), clindamycin (11), piperacillin/tazobactam (2), metronidazole (1), linezolid (1)	10–20 days	N = 66 DFI	-2% fungal intertrigo, 2% skin rash clindamycin, 2% toxic skin reaction due to A/C or levofloxacin, 2% cardiac decompensation due to P/T.

Footnote: Data are shown as % (CI 95%) for most results. Acronyms: RCT, randomized clinical trial; IT, interventional trial; Obs, observational study; RS, retrospective study; A/S, ampicillin/sulbactam; A/C, amoxicillin–clavulanate; A/V, aztreonam/vancomycin; CDI, Clostridium difficile; DFI, diabetic foot infections; DFU, diabetic foot ulcer; GI, gastrointestinal; I.V: intravenous; I/C, imipenem–cilastatine; P/T, piperacillin–tazobactam; STI, soft-tissue infection; T/C, ticarcillin–clavulanate; T/S, trimethoprim–sulfamethoxazole.

## 3. Discussion

Recurrent misuse of systemic antibiotics is high in the adult population with DFIs [6]. With long antibiotic treatments, the risk for antibiotic-related AEs may increase. Most systemic AEs occur during the first three weeks of treatment [19,63]. We have limited the review to the analysis of 53 selected articles looking for AEs documented in the published literature on DFI. Therefore, some AEs could be underrepresented if no articles were documented.

Overall, gastrointestinal intolerances were the most reported AE, ranging from 5% to 22% among all antibiotic therapies, although this occurred most frequently during oral beta-lactam or clindamycin use and at the increasing doses related to tetracyclines. Symptomatic colitis due to *Clostridium difficile* may be a severe complication and is higher for beta-lactams (8–9%) than other antibiotics. More serious AEs were reported: hepatotoxicity due to beta-lactams may not be as rare (5% to 17%) and it is important to be aware of that for monitoring care. The hepatotoxicity of fluoroquinolones is generally lower (3%), and there is cytopenia related to linezolid (5%) or beta-lactams (5% to 7%), nausea and multiple drug interactions under rifampicin, and renal insufficiency under cotrimoxazole.

Kidney function may be altered by a wide range of antibiotic agents. However, vancomycin (compared with other agents) showed a higher potential for reduced glomerular filtration, and monitoring levels for vancomycin could help to avoid toxicity for which a potential overdose may lead to dialysis. Furthermore, and despite that the selected studies could not prove this toxicity, the regular use of cotrimoxazole at high doses is associated with transient nephrotoxicity and metabolic acidosis [13] in serum laboratory controls.

Hematologic disturbances occur regularly with linezolid when prescribed for more than 10–14 days, with fewer cytopenia cases with tedizolid compared with linezolid. This mechanism of inhibition of bacterial ribosomes also has an effect on the mitochondrial protein synthesis of human cells and causes mitochondrial dysfunction with a decrease in ATP in the bone marrow precursor cells that leads to myelosuppression. This is important to know to fix the data to request a control hemogram blood test if treatment is to be continued for a longer period of time, i.e., more than 14 days. Since tedizolid is administered once a day, it allows longer recovery periods from mitochondrial dysfunction and can mitigate the higher intrinsic toxicity compared with linezolid [72].

Skin rash was shown to seldom occur and was typically related to use of penicillins or cotrimoxazole. We believe inguinal mycosis (intertrigo) is mostly recognized in only hospitalized patients and is therefore underreported in the literature. Severe cutaneous and systemic toxic reactions have been described, such as eosinophilic rash (DRESS syndrome) together with a systemic inflammatory response syndrome (SIRS) with beta-lactam drugs, clindamycin, and vancomycin. In addition, cotrimoxazole may also be a potential cause of SIRS. The Stevens–Johnson syndrome of severe skin toxicity is not described in our focused review on DFIs, but clinicians should also be aware of this severe complication of antibiotic therapy, it being more frequent with sulfonamides and beta-lactams.

All systemic antibiotic agents in diabetic patients with a poor glycemic control and classically with macerated inguinal regions due to overlapping abdominal fat tissues may produce fungal skin infections during or shortly after the antibiotic therapy (e.g., intertrigo). However, we found data regarding this clinically frequent observation to be sparce. One article attributed an overall incidence of 2% and 4% concerning all antibiotic classes [12,19], and in our Balgrist orthopedic cohort experience from 2018 to 2022, which included at least one AE in 250 patients from 2312 orthopedic infections including diabetic patients (737, 32%), skin mycosis affected 51 patients (2.2%) (personal communication IU and LSB; not published before).

The musculoskeletal system was an uncommonly described AE, despite the fact that the risk of tendinopathies is a hallmark of fluoroquinolones; however, the published literature was not related to that of the selected DFI articles. Regarding the risk of quinolone-related tendon pathology, it is reported in the literature as a rare adverse event in 0.5–2% of cases treated with fluoroquinolones [73].

With regards to neurological AEs, headache or insomnia is frequently described for many antibiotics. The incidences of such conditions are higher with quinolones, whereas seizures, confusions, or hallucinations are commonly associated with beta-lactam agents. Seizures are related to the beta-lactam ring, which binds to GABA receptors and alters ionic conduction. The most frequent neurological disturbances (i.e., somnolence or confusion) occur with 3rd–4th generation cephalosporins (high with cefepime) and imipenem. A switch to early oral therapy by other agents or a therapeutic drug monitoring of the serum cefepime level in elderly patients with impaired renal function can be an option to reduce neurological AEs in patients with DFI. In our review, within the low frequency of seizures due to carbapenems, imipenem was the most common causative agent. Of note, monobactams (aztreonam) are less involved in neurological events [74].

The main limitation of this review is the restriction to scientific publications in the English language only and to articles specifically targeting DFI antibiotic therapy. It is clear that the same antibiotic agents might represent other AE in other patient populations with fewer co-morbidities inherent to the DFI patient population. Such an expanded review would be beyond the scope of this article. Likewise, we excluded the problem of additional costs specifically related to the antibiotic-related AEs during the treatment of DFI (for which there are no specific data available) and we purposely skip the discussion of antimicrobial resistances in the management of DFI, which many clinicians would indicate as the most important adverse problem. However, the resistance problem is not a classical or random ‘adverse event’ of antibiotic therapy. It is a systematic flipside of each prolonged antibiotic administration in human and veterinary medicine. Articles on antimicrobial resistances in DFI treatment already exist and would complicate the content and structure of this manuscript.

## 4. Materials and Methods

### 4.1. Research Criteria

In October 2022 we searched PubMed using two distinct MeSH terms for studies relating to antibiotic-related AEs in DFI. We searched for relevant titles and abstracts with the search terms “antibiotics” AND “diabetic foot”. Our second search included the search terms “antibiotics” AND (“skin-structure infection” OR “soft tissue infection”) NOT (“skin grafting” OR “psoriasis” OR “tumor” OR “hematologic disease”). In both searches we included clinical studies, clinical trials, clinical trial protocols, multicenter studies, and randomized controlled trials that were published in the English language with adult patients 19 years and above between January 1990 and September 2022. Figure S1 shows our study selection flow diagram. Purposely, we excluded the problem of antimicrobial resistances linked to the (over)use of antibiotics, for which other and broader reviews are available.

We calculated 95% confidence intervals for proportions following the Clopper–Pearson approach.

In preparing this narrative review, we adhered to key applicable items for narrative reviews from the PRISMA statement [75].

### 4.2. Criteria for Considering Studies for This Review

We included randomized controlled trials (RCTs), nonrandomized or quasi-randomized controlled trials, and observational studies (prospective or retrospective observational epidemiological studies) from January 1990 to September 2022 in the English language. We included studies of people with diabetes mellitus (type 1 or 2) with any type of foot wound (ulcers of neuropathic or ischemic etiology) and with DFI (osteomyelitis or soft-tissue infections). All patients included in the selected studies had to be treated with systemic antibiotics (any type oral or parenteral) with well-described reports of adverse effects.

### 4.3. Exclusion Criteria

We excluded studies that evaluated infections other than soft-tissue infection or osteomyelitis at the same time as DFI, studies that lacked data on antibiotic-related AEs, studies that included a case series with less than 30 diabetic patients, and studies that reported on topical antibiotics.

## 5. Conclusions

There is a clear need for greater antibiotic stewardship in the clinical management of DFIs given the increasing worldwide incidence and the increased use of antibiotic therapies that lead to an increase the days of hospitalization and higher complications for patients. Epidemiological understanding of antibiotic-related AEs is considered the cornerstone of every stewardship program and cannot be limited to only the acquisition of resistances and costs. The medical complications that arise from some AEs are not negligible.

The proportion of AEs in patients during or shortly after antibiotic therapy for DFI was shown to be as high as 30%. The risks and benefits of prolonged antibiotic use must be considered in each clinical situation, and knowledge of the most common and most severe AEs is useful for the clinician to be aware of the presentation of these AEs. A proven method to protect against such AEs is to reduce the duration of antibiotic treatment with lowered dosages as deemed clinically necessary. As mentioned by the stewardship program, clinicians should have an understanding of possible AEs and inform patients in cases where longer antibiotic therapy is deemed necessary.

## Data Availability

Not applicable.

## Supplementary Materials

The following supporting information can be downloaded at: https://www.mdpi.com/article/10.3390/antibiotics12040774/s1, Table S1: Drug–drug interactions. Figure S1: Flowchart of selected studies.

## References

[1] Sen P., Demirdal T., Emir B. (2019). Meta-Analysis of Risk Factors for Amputation in Diabetic Foot Infections. Diabetes Metab. Res. Rev..

[2] Lebowitz D., Gariani K., Kressmann B., von Dach E., Huttner B., Bartolone P., Lê N., Mohamad M., Lipsky B.A., Uçkay I. (2017). Are Antibiotic-Resistant Pathogens More Common in Subsequent Episodes of Diabetic Foot Infection?. Int. J. Infect. Dis..

[3] Lipsky B.A., Berendt A.R., Cornia P.B., Pile J.C., Peters E.J.G., Armstrong D.G., Deery H.G., Embil J.M., Joseph W.S., Karchmer A.W. (2012). 2012 Infectious Diseases Society of America Clinical Practice Guideline for the Diagnosis and Treatment of Diabetic Foot Infectionsa. Clin. Infect. Dis..

[4] Senneville E., Melliez H., Beltrand E., Legout L., Valette M., Cazaubie M., Cordonnier M., Caillaux M., Yazdanpanah Y., Mouton Y. (2006). Culture of Percutaneous Bone Biopsy Specimens for Diagnosis of Diabetic Foot Osteomyelitis: Concordance with Ulcer Swab Cultures. Clin. Infect. Dis..

[5] Lipsky B.A., Senneville É., Abbas Z.G., Aragón-Sánchez J., Diggle M., Embil J.M., Kono S., Lavery L.A., Malone M., Asten S.A. (2020). Guidelines on the Diagnosis and Treatment of Foot Infection in Persons with Diabetes (IWGDF 2019 Update). Diabetes Metab. Res. Rev..

[6] Uçkay I., Berli M., Sendi P., Lipsky B.A. (2019). Principles and Practice of Antibiotic Stewardship in the Management of Diabetic Foot Infections. Curr. Opin. Infect. Dis..

[7] Driver V.R., Fabbi M., Lavery L.A., Gibbons G. (2010). The Costs of Diabetic Foot: The Economic Case for the Limb Salvage Team. J. Vasc. Surg..

[8] Beringer P.M., Wong-Berringer A., Rho J.P. (1998). Economic Aspects of Antibacterial Adverse Effects. Pharmacoeconomics.

[9] Henig O., Pogue J.M., Martin E., Hayat U., Ja’ara M., Kilgore P.E., Cha R., Dhar S., Kaye K.S. (2020). The Impact of Multidrug-Resistant Organisms on Outcomes in Patients with Diabetic Foot Infections. Open Forum. Infect. Dis..

[10] Uçkay I., Holy D., Betz M., Sauer R., Huber T., Burkhard J. (2021). Osteoarticular Infections: A Specific Program for Older Patients?. Aging Clin. Exp. Res..

[11] Selva Olid A., Solà I., Barajas-Nava L.A., Gianneo O.D., Bonfill Cosp X., Lipsky B.A. (2015). Systemic Antibiotics for Treating Diabetic Foot Infections. Cochrane Database Syst. Rev..

[12] Pham T.-T., Gariani K., Richard J.-C., Kressmann B., Jornayvaz F.R., Philippe J., Lipsky B.A., Uçkay I. (2022). Moderate to Severe Soft Tissue Diabetic Foot Infections. Ann. Surg..

[13] Grayson M.L., Crowe S.M., McCarthy J.S., Mills J., Mouton J.W., Paterson D.L., Norrby S.R., Pfaller M.A. (2017). Kucers’ The Use of Antibiotics.

[14] Legat F.J., Krause R., Zenahlik P., Hoffmann C., Scholz S., Salmhofer W., Tscherpel J., Tscherpel T., Kerl H., Dittrich P. (2005). Penetration of Piperacillin and Tazobactam into Inflamed Soft Tissue of Patients with Diabetic Foot Infection. Antimicrob. Agents Chemother..

[15] Uçkay I., Pires D., Agostinho A., Guanziroli N., Öztürk M., Bartolone P., Tscholl P., Betz M., Pittet D. (2017). Enterococci in Orthopaedic Infections: Who Is at Risk Getting Infected?. J. Infect..

[16] Zenelaj B., Bouvet C., Lipsky B.A., Uçkay I. (2014). Do Diabetic Foot Infections with Methicillin-Resistant *Staphylococcus Aureus* Differ from Those with Other Pathogens?. Int. J. Low. Extrem. Wounds.

[17] Garau J., Ostermann H., Medina J., Ávila M., McBride K., Blasi F. (2013). Current Management of Patients Hospitalized with Complicated Skin and Soft Tissue Infections across Europe (2010–2011): Assessment of Clinical Practice Patterns and Real-Life Effectiveness of Antibiotics from the REACH Study. Clin. Microbiol. Infect..

[18] Graham D.R., Talan D.A., Nichols R.L., Lucasti C., Corrado M., Morgan N., Fowler C.L. (2002). Once-Daily, High-Dose Levofloxacin versus Ticarcillin-Clavulanate Alone or Followed by Amoxicillin-Clavulanate for Complicated Skin and Skin-Structure Infections: A Randomized, Open-Label Trial. Clin. Infect. Dis..

[19] Gariani K., Pham T.-T., Kressmann B., Jornayvaz F.R., Gastaldi G., Stafylakis D., Philippe J., Lipsky B.A., Uçkay L. (2021). Three Weeks Versus Six Weeks of Antibiotic Therapy for Diabetic Foot Osteomyelitis: A Prospective, Randomized, Noninferiority Pilot Trial. Clin. Infect. Dis..

[20] Chantelau E., Tanudjaja T., Altenhöfer F., Ersanli Z., Lacigova S., Metzger C. (1996). Antibiotic Treatment for Uncomplicated Neuropathic Forefoot Ulcers in Diabetes: A Controlled Trial. Diabet. Med..

[21] Tan J.S., Wishnow R.M., Talan D.A., Duncanson F.P., Norden C.W. (1993). Treatment of Hospitalized Patients with Complicated Skin and Skin Structure Infections: Double-Blind, Randomized, Multicenter Study of Piperacillin-Tazobactam versus Ticarcillin-Clavulanate. The Piperacillin/Tazobactam Skin and Skin Structure Study Group. Antimicrob. Agents Chemother..

[22] Akova M., Özcebe O., Güllü I., Ünal S., Gür D., Akalin S., Tokgözoglu M., Telatar F., Akalin H.E. (1996). Efficacy of Sulbactam-Ampicillin for the Treatment of Severe Diabetic Foot Infections. J. Chemother..

[23] Lipsky B.A., Armstrong D.G., Citron D.M., Tice A.D., Morgenstern D.E., Abramson M.A. (2005). Ertapenem versus Piperacillin/Tazobactam for Diabetic Foot Infections (SIDESTEP): Prospective, Randomised, Controlled, Double-Blinded, Multicentre Trial. Lancet.

[24] Gyssens I.C., Dryden M., Kujath P., Nathwani D., Schaper N., Hampel B., Reimnitz P., Alder J., Arvis P. (2011). A Randomized Trial of the Efficacy and Safety of Sequential Intravenous/Oral Moxifloxacin Monotherapy versus Intravenous Piperacillin/Tazobactam Followed by Oral Amoxicillin/Clavulanate for Complicated Skin and Skin Structure Infections. J. Antimicrob. Chemother..

[25] McKinnon P.S., Paladino J.A., Grayson M.L., Gibbons G.W., Karchmer A.W. (1997). Cost-Effectiveness of Ampicillin/Sulbactam Versus Imipenem/Cilastatin in the Treatment of Limb-Threatening Foot Infections in Diabetic Patients. Clin. Infect. Dis..

[26] Grayson M.L., Gibbons G.W., Habershaw G.M., Freeman D.V., Pomposelli F.B., Rosenblum B.I., Levin E., Karchmer A.W. (1994). Use of Ampicillin/Sulbactam Versus Imipenem/Cilastatin in the Treatment of Limb-Threatening Foot Infections in Diabetic Patients. Clin. Infect. Dis..

[27] Harkless L., Boghossian J., Pollak R., Caputo W., Dana A., Gray S., Wu D. (2005). An Open-Label, Randomized Study Comparing Efficacy and Safety of Intravenous Piperacillin/Tazobactam and Ampicillin/Sulbactam for Infected Diabetic Foot Ulcers. Surg. Infect..

[28] Saltoglu N., Dalkiran A., Tetiker T., Bayram H., Tasova Y., Dalay C., Sert M. (2010). Piperacillin/Tazobactam versus Imipenem/Cilastatin for Severe Diabetic Foot Infections: A Prospective, Randomized Clinical Trial in a University Hospital. Clin. Microbiol. Infect..

[29] Graham D.R., Lucasti C., Malafaia O., Nichols R.L., Holtom P., Perez N.Q., McAdams A., Woods G.L., Ceesay T.P., Gesser R. (2002). Ertapenem Once Daily Versus Piperacillin-Tazobactam 4 Times per Day for Treatment of Complicated Skin and Skin-Structure Infections in Adults: Results of a Prospective, Randomized, Double-Blind Multicenter Study. Clin. Infect. Dis..

[30] Stengel D., Görzer E., Schintler M., Legat F.J., Amann W., Pieber T., Ekkernkamp A., Graninger W. (2005). Second-Line Treatment of Limb-Threatening Diabetic Foot Infections with Intravenous Fosfomycin. J. Chemother..

[31] Schwartz R., Das-Young L.R., Ramirez-Ronda C., Frank E. (1996). Current and Future Management of Serious Skin and Skin-Structure Infections. Am. J. Med..

[32] Corey G.R., Wilcox M., Talbot G.H., Friedland H.D., Baculik T., Witherell G.W., Critchley I., Das A.F., Thye D. (2010). Integrated Analysis of CANVAS 1 and 2: Phase 3, Multicenter, Randomized, Double-Blind Studies to Evaluate the Safety and Efficacy of Ceftaroline versus Vancomycin plus Aztreonam in Complicated Skin and Skin-Structure Infection. Clin. Infect. Dis..

[33] Santos P.D., Davis A., Jandourek A., Smith A., David Friedland H. (2013). Ceftaroline Fosamil and Treatment of Acute Bacterial Skin and Skin Structure Infections: CAPTURE Study Experience. J. Chemother..

[34] Lipsky B.A., Cannon C.M., Ramani A., Jandourek A., Calmaggi A., Friedland H.D., Goldstein E.J.C. (2015). Ceftaroline Fosamil for Treatment of Diabetic Foot Infections: The CAPTURE Study Experience. Diabetes/Metab. Res. Rev..

[35] Noel G.J., Bush K., Bagchi P., Ianus J., Strauss R.S. (2008). A Randomized, Double-Blind Trial Comparing Ceftobiprole Medocaril with Vancomycin plus Ceftazidime for the Treatment of Patients with Complicated Skin and Skin-Structure Infections. Clin. Infect. Dis..

[36] Embil J.M., Soto N.E., Melnick D.A. (2006). A Post Hoc Subgroup Analysis of Meropenem versus Imipenem/Cilastatin in a Multicenter, Double-Blind, Randomized Study of Complicated Skin and Skin-Structure Infections in Patients with Diabetes Mellitus. Clin. Ther..

[37] Xu Z.R., Ran X.W., Xian Y., Yan X.D., Yuan G.Y., Mu S.M., Shen J.F., Zhang B.S., Gan W.J., Wang J. (2016). Ertapenem versus Piperacillin/Tazobactam for Diabetic Foot Infections in China: A Phase 3, Multicentre, Randomized, Double-Blind, Active-Controlled, Non-Inferiority Trial. J. Antimicrob. Chemother..

[38] Fabian T.C., File T.M., Embil J.M., Krige J.E.J., Klein S., Rose A., Melnick D., Soto N.E. (2005). Meropenem Versus Imipenem-Cilastatin for the Treatment of Hospitalized Patients with Complicated Skin and Skin Structure Infections: Results of a Multicenter, Randomized, Double-Blind Comparative Study. Surg. Infect..

[39] Giordano P., Song J., Pertel P., Herrington J., Kowalsky S. (2005). Sequential Intravenous/Oral Moxifloxacin versus Intravenous Piperacillin-Tazobactam Followed by Oral Amoxicillin-Clavulanate for the Treatment of Complicated Skin and Skin Structure Infection. Int. J. Antimicrob. Agents.

[40] Vick-Fragoso R., Hernández-Oliva G., Cruz-Alcázar J., Amábile-Cuevas C.F., Arvis P., Reimnitz P., Bogner J.R. (2009). Efficacy and Safety of Sequential Intravenous/Oral Moxifloxacin vs Intravenous/Oral Amoxicillin/Clavulanate for Complicated Skin and Skin Structure Infections. Infection.

[41] Bogner J.R., Kutaiman A., Esguerra-Alcalen M., Heldner S., Arvis P. (2013). Moxifloxacin in Complicated Skin and Skin Structure Infections (CSSSIs): A Prospective, International, Non-Interventional, Observational Study. Adv. Ther..

[42] Lipsky B.A., Giordano P., Choudhri S., Song J. (2007). Treating Diabetic Foot Infections with Sequential Intravenous to Oral Moxifloxacin Compared with Piperacillin-Tazobactam/Amoxicillin-Clavulanate. J. Antimicrob. Chemother..

[43] Lipsky B.A., Baker P.D., Landon G.C., Fernau R. (1997). Antibiotic Therapy for Diabetic Foot Infections: Comparison of Two Parenteral-to-Oral Regimens. Clin. Infect. Dis..

[44] Segev S., Rosen N., Pitlik S.D., Block C., Rubinstein E. (1990). Pefloxacin versus Ceftazidime in Therapy of Soft Tissue Infections in Compromised Patients. J. Antimicrob. Chemother..

[45] Siami F.S., LaFleur B.J., Siami G.A. (2002). Clinafloxacin versus Piperacillin/Tazobactam in the Treatment of Severe Skin and Soft-Tissue Infections in Adults at a Veterans Affairs Medical Center. Clin. Ther..

[46] Lipsky B.A., Miller B., Schwartz R., Henry D.C., Nolan T., McCabe A., Magner D.J., Talbot G.H. (1999). Sparfloxacin versus Ciprofloxacin for the Treatment of Community-Acquired, Complicated Skin and Skin-Structure Infections. Clin. Ther..

[47] Schaper N.C., Dryden M., Kujath P., Nathwani D., Arvis P., Reimnitz P., Alder J., Gyssens I.C. (2013). Efficacy and Safety of IV/PO Moxifloxacin and IV Piperacillin/Tazobactam Followed by PO Amoxicillin/Clavulanic Acid in the Treatment of Diabetic Foot Infections: Results of the RELIEF Study. Infection.

[48] Bassetti M., Righi E., Pecori D., Tillotson G. (2018). Delafloxacin: An Improved Fluoroquinolone Developed through Advanced Molecular Engineering. Future Microbiol..

[49] Li C., Mercuro N.J., Chapin R.W., Gold H.S., McCoy C. (2021). Fluoroquinolone Prescribing for Diabetic Foot Infections Following an FDA Drug Safety Communication for Aortic Aneurysm Risk. Antimicrob. Agents Chemother..

[50] Bhatia A., Mastim M., Shah M., Gutte R., Joshi P., Kumbhar D., Periasamy H., Palwe S.R., Chavan R., Bhagwat S. (2020). Efficacy and Safety of a Novel Broad-Spectrum Anti-MRSA Agent Levonadifloxacin Compared with Linezolid for Acute Bacterial Skin and Skin Structure Infections: A Phase 3, Openlabel, Randomized Study. J. Assoc. Physicians India.

[51] Tone A., Nguyen S., Devemy F., Topolinski H., Valette M., Cazaubiel M., Fayard A., Beltrand É., Lemaire C., Senneville É. (2015). Six-Week Versus Twelve-Week Antibiotic Therapy for Nonsurgically Treated Diabetic Foot Osteomyelitis: A Multicenter Open-Label Controlled Randomized Study. Diabetes Care.

[52] Itani K.M.F., Weigelt J., Li J.Z., Duttagupta S. (2005). Linezolid Reduces Length of Stay and Duration of Intravenous Treatment Compared with Vancomycin for Complicated Skin and Soft Tissue Infections Due to Suspected or Proven Methicillin-Resistant Staphylococcus Aureus (MRSA). Int. J. Antimicrob. Agents.

[53] Chuang Y.C., Chang C.M., Aradhya S., Nagari B., Pai V., Dartois N., Jouve S., Cooper A. (2011). Efficacy and Safety of Tigecycline Monotherapy Compared with Vancomycin-Aztreonam in the Treatment of Complicated Skin and Skin Structure Infections in Patients from India and Taiwan. J. Microbiol. Immunol. Infect..

[54] Itani K.M.F., Dryden M.S., Bhattacharyya H., Kunkel M.J., Baruch A.M., Weigelt J.A. (2010). Efficacy and Safety of Linezolid versus Vancomycin for the Treatment of Complicated Skin and Soft-Tissue Infections Proven to Be Caused by Methicillin-Resistant Staphylococcus Aureus. Am. J. Surg..

[55] Trinh T.D., Jorgensen S.C.J., Zasowski E.J., Claeys K.C., Lagnf A.M., Estrada S.J., Delaportes D.J., Huang V., Klinker K.P., Kaye K.S. (2019). Multicenter Study of the Real-World Use of Ceftaroline versus Vancomycin for Acute Bacterial Skin and Skin Structure Infections. Antimicrob. Agents Chemother..

[56] Arbeit R.D., Maki D., Tally F.P., Campanaro E., Eisenstein B.I. (2004). The Safety and Efficacy of Daptomycin for the Treatment of Complicated Skin and Skin-Structure Infections. Clin. Infect. Dis..

[57] Lipsky B.A., Stoutenburgh U. (2005). Daptomycin for Treating Infected Diabetic Foot Ulcers: Evidence from a Randomized, Controlled Trial Comparing Daptomycin with Vancomycin or Semi-Synthetic Penicillins for Complicated Skin and Skin-Structure Infections. J. Antimicrob. Chemother..

[58] Rappo U., Gonzalez P.L., Puttagunta S., Akinapelli K., Keyloun K., Gillard P., Liu Y., Dunne M.W. (2019). Single-Dose Dalbavancin and Patient Satisfaction in an Outpatient Setting in the Treatment of Acute Bacterial Skin and Skin Structure Infections. J. Glob. Antimicrob. Resist..

[59] Boucher H.W., Wilcox M., Talbot G.H., Puttagunta S., Das A.F., Dunne M.W. (2014). Once-Weekly Dalbavancin versus Daily Conventional Therapy for Skin Infection. N. Engl. J. Med..

[60] Corey G.R., Good S., Jiang H., Moeck G., Wikler M., Green S., Manos P., Keech R., Singh R., Heller B. (2015). Single-Dose Oritavancin Versus 7–10 Days of Vancomycin in the Treatment of Gram-Positive Acute Bacterial Skin and Skin Structure Infections: The SOLO II Noninferiority Study. Clin. Infect. Dis..

[61] Lipsky B.A., Itani K., Norden C. (2004). Treating Foot Infections in Diabetic Patients: A Randomized, Multicenter, Open-Label Trial of Linezolid versus Ampicillin-Sulbactam/Amoxicillin-Clavulanate. Clin. Infect. Dis..

[62] Mikamo H., Takesue Y., Iwamoto Y., Tanigawa T., Kato M., Tanimura Y., Kohno S. (2018). Efficacy, Safety and Pharmacokinetics of Tedizolid versus Linezolid in Patients with Skin and Soft Tissue Infections in Japan—Results of a Randomised, Multicentre Phase 3 Study. J. Infect. Chemother..

[63] Uçkay I., Kressmann B., Malacarne S., Toumanova A., Jaafar J., Lew D., Lipsky B.A. (2018). A Randomized, Controlled Study to Investigate the Efficacy and Safety of a Topical Gentamicin-Collagen Sponge in Combination with Systemic Antibiotic Therapy in Diabetic Patients with a Moderate or Severe Foot Ulcer Infection. BMC Infect. Dis..

[64] Uçkay I., Kressmann B., di Tommaso S., Portela M., Alwan H., Vuagnat H., Maître S., Paoli C., Lipsky B.A. (2018). A Randomized Controlled Trial of the Safety and Efficacy of a Topical Gentamicin–Collagen Sponge in Diabetic Patients with a Mild Foot Ulcer Infection. SAGE Open Med..

[65] Teras J., Gardovskis J., Vaasna T., Kupcs U., Pupelis G., Dartois N., Jouve S., Cooper A. (2008). Overview of Tigecycline Efficacy and Safety in the Treatment of Complicated Skin and Structure Infections—A European Perspective. J. Chemother..

[66] Ellis-Grosse E.J., Babinchak T., Dartois N., Rose G., Loh E. (2005). The Efficacy and Safety of Tigecycline in the Treatment of Skin and Skin-Structure Infections: Results of 2 Double-Blind Phase 3 Comparison Studies with Vancomycin-Aztreonam. Clin. Infect. Dis..

[67] Lauf L., Ozsvár Z., Mitha I., Regöly-Mérei J., Embil J.M., Cooper A., Sabol M.B., Castaing N., Dartois N., Yan J. (2014). Phase 3 Study Comparing Tigecycline and Ertapenem in Patients with Diabetic Foot Infections with and without Osteomyelitis. Diagn. Microbiol. Infect. Dis..

[68] Abrahamian F.M., Sakoulas G., Tzanis E., Manley A., Steenbergen J., Das A.F., Eckburg P.B., McGovern P.C. (2019). Omadacycline for Acute Bacterial Skin and Skin Structure Infections. Clin. Infect. Dis..

[69] Montravers P., Bassetti M., Dupont H., Eckmann C., Heizmann W.R., Guirao X., Garcia M.S., Capparella M.R., Simoneau D., Bodmann K.F. (2013). Efficacy of Tigecycline for the Treatment of Complicated Skin and Soft-Tissue Infections in Real-Life Clinical Practice from Five European Observational Studies. J. Antimicrob. Chemother..

[70] Moran G.J., Krishnadasan A., Mower W.R., Abrahamian F.M., LoVecchio F., Steele M.T., Rothman R.E., Karras D.J., Hoagland R., Pettibone S. (2017). Effect of Cephalexin Plus Trimethoprim-Sulfamethoxazole vs Cephalexin Alone on Clinical Cure of Uncomplicated Cellulitis. JAMA.

[71] Lipsky B.A. (1990). Outpatient Management of Uncomplicated Lower-Extremity Infections in Diabetic Patients. Arch. Intern. Med..

[72] Flanagan S., McKee E.E., Das D., Tulkens P.M., Hosako H., Fiedler-Kelly J., Passarell J., Radovsky A., Prokocimer P. (2015). Nonclinical and Pharmacokinetic Assessments To Evaluate the Potential of Tedizolid and Linezolid to Affect Mitochondrial Function. Antimicrob. Agents Chemother..

[73] Kirchgesner T., Larbi A., Omoumi P., Malghem J., Zamali N., Manelfe J., Lecouvet F., Vande Berg B., Djebbar S., Dallaudière B. (2014). Drug-Induced Tendinopathy: From Physiology to Clinical Applications. Jt. Bone Spine.

[74] Deshayes S., Coquerel A., Verdon R. (2017). Neurological Adverse Effects Attributable to β-Lactam Antibiotics: A Literature Review. Drug Saf..

[75] Page M.J., McKenzie J.E., Bossuyt P.M., Boutron I., Hoffmann T.C., Mulrow C.D., Shamseer L., Tetzlaff J.M., Akl E.A., Brennan S.E. (2021). The PRISMA 2020 Statement: An Updated Guideline for Reporting Systematic Reviews. BMJ.

