# Supplementary material for: The Epidemiology of Antibiotic-Related Adverse Events in the Treatment of Diabetic Foot Infections: A Narrative Review of the Literature
Source: Antibiotics (Basel). 2023 Apr 18;12(4):774. doi: 10.3390/antibiotics12040774 (PMC10135215; doi:10.3390/antibiotics12040774)
Supplement: Supplementary file 1 [file antibiotics-12-00774-s001.zip › antibiotics-2336218-supplementary.pdf]

Supplementary Table S1. Drug–drug interactions.

Antibiotic	**Drug interaction**	**Adverse event**	**Management recommendations**
**Beta-lactams**	• Methotrexate<br>• Allopurinol<br>• Phenprocoumon	• Increased serum concentration of methotrexate<br>• Enhance the potential for allergic or hypersensitivity reactions to amoxicillin<br>• Increased risk of bleeding	• Monitor for toxic effects of methotrexate<br>• Monitor for an increased incidence of hypersensitivity reactions<br>• Close INR monitoring
**Quinolones**	• Multivitamins/minerals with polyvalent cations (e.g., magnesium, calcium, iron, zinc), antacids<br>• QT-prolonging agents, e.g., antidepressants, anti-psychotics, antiarrhythmics<br>• Corticosteroids (systemic)<br>• Phenprocoumon	• Reduce absorption of quinolones, risk of reduced bioavailability and efficacy<br>• Increased risk of QTc interval prolongation<br>• Enhance the adverse/toxic effect of quinolones, especially, the risk of tendonitis and tendon rupture may be increased.<br>• Increased risk of bleeding	• Consider administering an oral quinolone at least 2 hours before the dose of a multivitamin that contains polyvalent cations or an antacids<br>• EKG monitoring<br>• Monitor closely for new onset tendon or joint pain<br>• Monitor INR closely and consider reducing the vitamin K antagonist
**Glycopeptides, lipopeptides, and lipoglycopeptides**	• Statins	• Enhance the adverse/toxic effect of daptomycin	• Consider CPK monitoring or temporarily stopping statin therapy
**Oxazolidinones**	• SSRIs/SNRIs<br>Tripants<br>Tricyclic Antidepressants	• Risk of serotonin syndrome	• Avoid combination

**Tetracyclines**	• Multivitamins/minerals with polyvalent cations (e.g., magnesium, calcium, iron, zinc), antacids<br><br>• Phenprocoumon	• Reduce absorption of Tetracyclines, risk of reduced bioavailability and efficacy of Tetracyclines<br><br>• Risk of bleeding	• Consider administering an oral tetracycline at least 2 hours before the dose of a multivitamin that contains polyvalent cations or an antacid<br><br>• Monitor INR closely and consider reducing the Vitamin K Antagonist
**Cotrimoxazole**	• ACE-inhibitors Angiotensin II Receptor Blockers Spironolactone<br>• Phenprocoumon<br><br>• Antidiabetic Agents	• Hyperkalemia<br><br>• Increased risk of bleeding<br><br>• Enhance the hypoglycemic effect	• Close monitoring of serum potassium<br><br>• Monitor INR closely and consider reducing the vitamin K antagonist<br><br>• Observe closely hypoglycemic effects
**Rifampicin**	• Numerous interacting drugs, e.g., DOACs<br>• Statins, amitriptyline, citalopram	• Decreased serum concentration of the interacting drug	• Monitor and dose adjustment of the interacting drug

Footnote: INR: international normalized ratio blood test, CPK: creatine phosphokinase, ACE-inhibitors: angiotensin converting enzyme-inhibitor, DOACs: direct oral anticoagulants, SSRIs: selective serotonin reuptake inhibitors, SNRIs: serotonin and norepinephrine reuptake inhibitors.

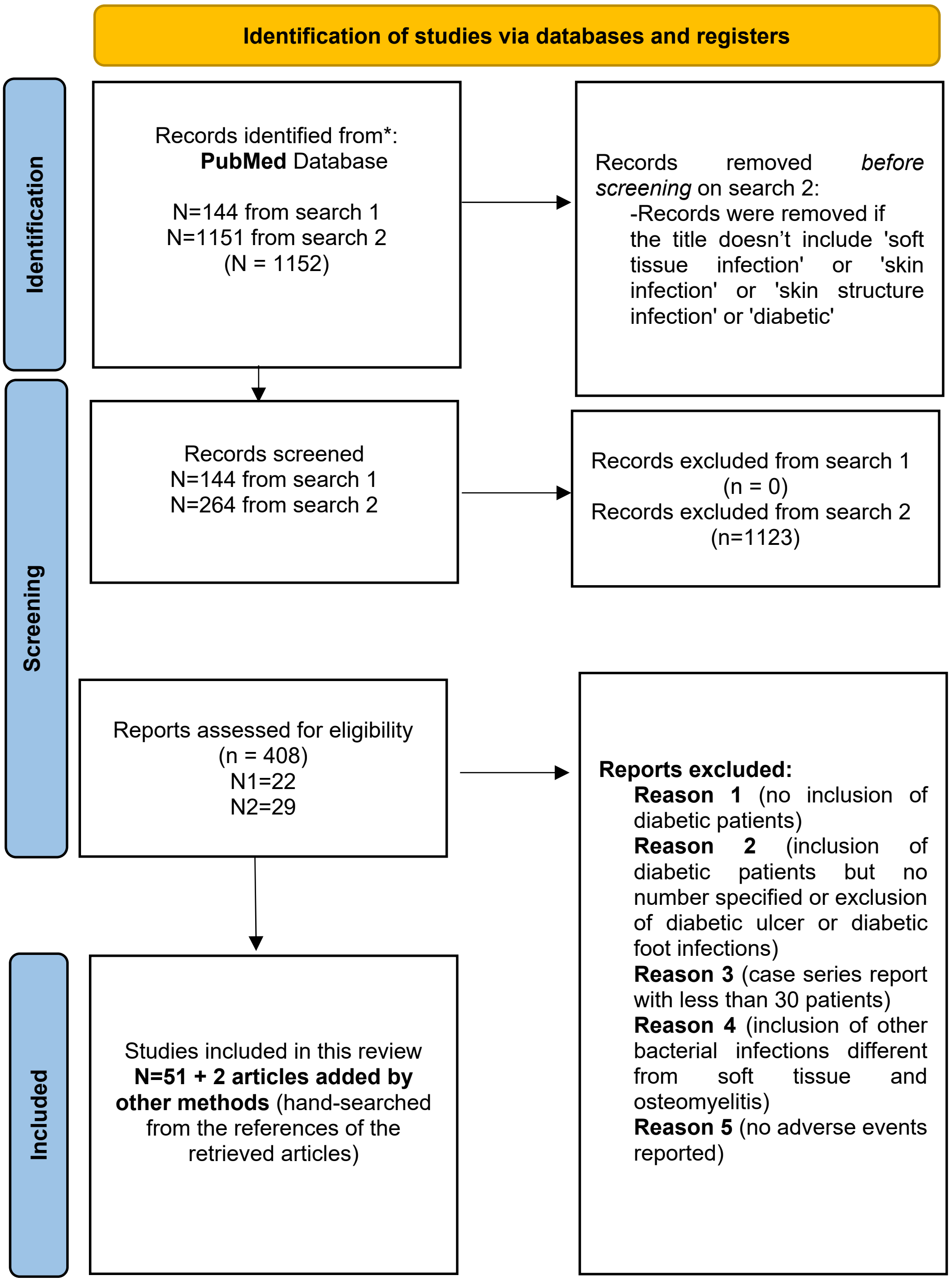

Figure S1. Flowchart of selected studies.
